# Comparison of AccuPower Diarrhea V1&V2 RT-PCR to a Chromatographic Immunoassay for Detecting Viral Pathogens from Human Diarrheal Stool Specimens

**DOI:** 10.3390/tropicalmed10020033

**Published:** 2025-01-24

**Authors:** Luka Katic, Boris Mihaljevic, Marijo Pirija, Ivana Goic-Barisic, Marija Tonkic, Anita Novak

**Affiliations:** 1 Department of Medicine, Icahn School of Medicine at Mount Sinai Morningside/West, 1000 Tenth Avenue, New York, NY 10019, USA; luka.katic@mountsinai.org; 2 ESCMID Food- and Water-borne Infections Study Group (EFWISG), 4051 Basel, Switzerland; 3 Department of Clinical Microbiology, University Hospital of Split, 21000 Split, Croatia; 4 School of Medicine Split, University of Split, 21000 Split, Croatia

**Keywords:** RT-PCR, chromatographic immunoassay, gastrointestinal viruses, diagnostic, sapovirus

## Abstract

Viruses are a frequent cause of self-limited diarrhea, with more severe outcomes in immunocompromised patients. This study aimed to compare the performance of Real-Time RT-PCR to chromatographic immunoassays (CIAs) for detecting the major gastrointestinal viruses in human stool. This study was conducted at the University Hospital of Split, Croatia, from October 2023 to May 2024. Stool samples were simultaneously analyzed with CIA (Acro Biotech Rotavirus and Adenovirus Combo Rapid Test Cassette, USA and JusChek Norovirus Rapid Test Cassette, China) and Real-Time RT-PCR (AccuPower Diarrhea V1&V2 Real-Time RT-PCR, Bioneer, Republic of Korea), according to the manufacturers’ instructions. Positive percent agreement (PPA), negative percent agreement (NPA), and overall percent agreement (OPA) were calculated. For norovirus, CIA had a low PPA (25%), indicating that it missed 75% of norovirus-positive cases identified by RT-PCR. Adenovirus detection by CIA showed poor agreement with RT-PCR (PPA 0%; NPA 100%). Rotavirus detection presented a relatively better performance with CIA (PPA 90.9% and OPA 84.13%). However, the presence of false positives (15.8%) highlights the need for confirmatory RT-PCR testing. One specimen was sapovirus-RT-PCR-positive, marking the first documented case from human specimens in Croatia. Although CIA provided rapid results, limitations regarding reliability highlight the value of RT-PCR, particularly in the case of ambiguous clinical cases with negative antigenic test results and newly emerged viruses. A two-step diagnostic approach, with initial CIA screening followed by confirmatory RT-PCR, could balance cost-effectiveness with diagnostic accuracy.

## 1. Introduction

Acute gastroenteritis (AGE) is one of the most common infectious diseases globally, affecting individuals across all age groups but disproportionately impacting young children and immunocompromised populations. AGE is characterized by symptoms such as diarrhea, vomiting, and abdominal pain, which can lead to significant morbidity and mortality, especially in resource-limited settings [1]. While mortality due to AGE has decreased in high-income countries due to advancements in healthcare and the introduction of vaccines, particularly for rotavirus, it remains a leading cause of child mortality in low-income regions [2]. Globally, rotavirus, norovirus, sapovirus, adenovirus, and astrovirus are among the most frequently identified viral agents causing AGE, each contributing to unique clinical and epidemiological patterns [3].

Rotaviruses (RVs), members of the Reoviridae family, are non-enveloped, double-stranded RNA viruses classified into 10 different species (A–J), with Rotavirus A being the leading cause of severe gastroenteritis in children under five years of age. They are responsible for over 200,000 deaths annually, predominantly in low-income countries. RV infects enterocytes in the small intestine, inducing diarrhea through mechanisms such as malabsorption due to enterocyte destruction, intestinal secretion stimulated by its enterotoxin (NSP4), and activation of the enteric nervous system. Despite widespread vaccination efforts since 2006, the effectiveness of live attenuated oral vaccines like Rotarix and RotaTeq remains suboptimal in low-income countries, likely due to factors such as environmental enteropathy and co-administration with other vaccines. Rotavirus infection can also manifest extra-intestinally, with potential associations with conditions like encephalitis and biliary atresia, though its systemic effects are not fully understood [4].

Norovirus (NoV) is a non-segmented, positive-stranded RNA virus and a member of the Caliciviridae family. It has only a single species, Norwalk virus, which is divided into six genogroups and 30 genotypes. NoV is a highly infectious gastrointestinal virus, requiring as few as 10 viral particles to initiate infection, and spreads through fecal–oral transmission, contaminated food or water, and contact with infected individuals. Therefore, norovirus has worldwide distribution, frequently causing outbreaks in healthcare facilities, schools, and the food service industry. Genogroup II genotype 4 (GII.4) strains dominate outbreaks due to their ability to rapidly evolve and escape herd immunity. While infections are typically self-limiting in healthy individuals, they can lead to severe complications in immunocompromised individuals, the elderly, and young children. Despite advances in understanding its pathogenesis, there is still no licensed vaccine available [5,6,7,8]. Effective prevention strategies include rehydration therapy and hygiene measures, with ondansetron showing promise as an adjunct to oral rehydration by reducing vomiting, though it may increase diarrhea temporarily [9].

Sapoviruses (SaVs) are members of the Caliciviridae family and are emerging as significant causes of AGE, particularly in young children. First identified in an orphanage in Sapporo, Japan, SaVs are non-enveloped, positive-sense, single-stranded RNA viruses classified into five genogroups, with GI, GII, GIV, and GV infecting humans. Globally, SaVs have a pooled prevalence of 3.4% among children with AGE, with genogroup I (GI) and genogroup II (GII) strains being predominant. SaV infections are often associated with milder symptoms compared to norovirus or rotavirus. Given the increasing prevalence of SaVs and their significant role in NoV-negative AGE cases, there is a growing need for improved surveillance and vaccine development targeting the most prevalent genotypes [10,11,12,13].

Human adenoviruses (HAdVs), members of the Adenoviridae family, are non-enveloped, double-stranded DNA viruses known to cause a variety of illnesses, including respiratory infections, conjunctivitis, and gastroenteritis. Among the seven recognized species (A–G), species F, particularly HAdV-F40 and HAdV-F41, is primarily associated with AGE. These enteric adenoviruses are the third leading cause of diarrhea-related mortality in children under five, following rotavirus and *Shigella*. The global prevalence of HAdV-associated gastroenteritis has shown an increasing trend, especially in post-2010 studies, and is most pronounced in regions with poor sanitation and malnutrition, such as Africa. Enteric adenoviruses are highly prevalent in children younger than five years due to their underdeveloped immunity. Despite these findings, there are significant gaps in the epidemiology of HAdVs, particularly regarding species and genotype distribution [14].

Human astroviruses (HAstVs) are non-enveloped, positive-sense, single-stranded RNA viruses that belong to the Astroviridae family, which is classified into classic and novel genotypes. Transmission occurs via the fecal–oral route, with infections often presenting as mild, self-limiting diarrhea, though severe cases can occur in immunocompromised individuals. Molecular methods such as RT-PCR and nested RT-PCR are highly sensitive for detecting HAstVs, which are frequently identified in co-infections with other gastrointestinal viruses, like rotavirus and norovirus. Despite their widespread prevalence, research into effective vaccines or targeted therapeutic interventions for HAstVs remains limited [15].

Traditionally, the detection of gastrointestinal (GI) viruses in clinical laboratories has relied on enzyme immunoassays (EIAs) and chromatographic immunoassays (CIAs), which are rapid but often lack sensitivity and specificity, and cannot detect all gastrointestinal viruses, such as sapovirus [1,16,17]. More sensitive methods, like cultivation and electron microscopy, are reserved for referral laboratories and are inconvenient for everyday practice. The advent of molecular diagnostic techniques, particularly multiplex real-time reverse transcription polymerase chain reaction (RT-PCR) assays, has revolutionized pathogen detection by enabling simultaneous identification of multiple viruses with superior accuracy [18,19,20,21]. Advances in multiplex RT-PCR panels provide superior accuracy, allowing simultaneous detection of multiple pathogens with high sensitivity and specificity, making them valuable for outbreak management and improving healthcare practices [1,22].

Emerging diagnostic platforms, like the FilmArray GI Panel and xTAG GPP, demonstrate high accuracy in identifying gastrointestinal pathogens, including mixed infections and underdiagnosed viruses like sapovirus and astrovirus [23,24]. Molecular methods also facilitate the genotyping and surveillance of evolving strains, as seen with norovirus GII.4 variants, which develop through antigenic drift [25]. However, high costs, infrastructure requirements, and challenges in interpreting co-detections limit their routine use [26,27]. To optimize clinical utility, a two-step approach combining rapid antigen testing with confirmatory RT-PCR is recommended [27,28].

Despite these advancements, there remains limited evidence on the clinical utility of these assays compared to traditional methods in routine diagnostic settings. This study aimed to compare the performance of the AccuPower Diarrhea V1&V2 Real-Time RT-PCR, Bioneer (Daejeon, Republic of Korea), with CIA for detecting major GI viruses in stool specimens. Additionally, this study sought to evaluate the diagnostic reliability and clinical applicability of these methods, and to document the first confirmed case of sapovirus in Croatia.

## 2. Materials and Methods

### 2.1. Specimen Collection

This study was conducted at the Department of Clinical Microbiology, University Hospital of Split, Croatia, over a period of seven months (October 2023 to May 2024). Samples of diarrheal stool with a clinical request for virus detection were tested with CIA, as a part of a routine laboratory diagnostic. Working diagnoses were made by the physicians, according to the World Health Organization (WHO) (https://www.who.int/classifications/classification-of-diseases, accessed on 8 January 2025).

All samples with positive results for at last two GI viruses (suspected cross-reactivity) and specimens from immunocompromised patients (regardless of CIA result) were further tested with RT-PCR, as a confirmatory method, according to laboratory protocol. A total of 64 stool samples from patients presenting with acute diarrhea were collected and analyzed. Samples were collected from inpatients and outpatients within a maximum of 2 h after collection, tested immediately with CIA, and then stored at −80 °C until RT-PCR analysis was performed.

Demographic and clinical data, including patient age, gender, diagnosis, and hospital status (inpatient or outpatient), were recorded. One sample was excluded from the analysis due to invalid RT-PCR results, leaving a total of 63 samples for evaluation.

### 2.2. Chromatographic Immunoassay (CIA) and Real-Time RT-PCR

#### 2.2.1. Chromatographic Immunoassay (CIA)

For antigen detection, rapid CIA tests were used for rotavirus, adenovirus, and norovirus, according to the manufacturers’ protocol. The Acro Biotech Rotavirus and Adenovirus Combo Rapid Test Cassette (Feces) USA (Acro Biotech, Inc., Rancho Cucamonga, CA, USA), a lateral flow chromatographic immunoassay, was used to simultaneously detect rotavirus and adenovirus, providing qualitative results within 10 min. This is a rapid, lateral flow, chromatographic, one-step immunoassay for the qualitative detection of rotavirus and adenovirus in human feces specimens to aid in the diagnosis of rotavirus or adenovirus infection. In this test, the membrane is pre-coated with anti-rotavirus antibody and anti-adenovirus antibody on different regions of the test. During testing, a mixture of the specimen and reagent migrates upward on the membrane chromatographically by capillary action to react with the anti-rotavirus and anti-adenovirus antibodies on the membrane and generate a colored line. An internal procedural control is included in the test. According to the manufacturer, the test has been previously compared with the unknown latex agglutination method, demonstrating an overall accuracy of ≥97.0%.

The JusChek Norovirus Rapid Test Cassette (Hangzhou AllTest Biotech Co., Ltd., Hangzhou, China), was used to detect norovirus genogroups I and II. This is a rapid, lateral flow, chromatographic immunoassay for the qualitative detection of norovirus in human feces specimen to aid in the diagnosis of norovirus infection. The test utilizes monoclonal antibodies specific for norovirus genogroup I and II, coated on the test membrane, to selectively detect norovirus from human feces specimens. During testing, the stool specimen reacts with the conjugate antibodies; the mixture migrates upward on the membrane chromatographically by capillary action to react with norovirus antibodies on the membrane. An internal procedural control is included in the test. A limitation is that stool samples from infants under one year old can produce a false positive result. The performance of this test has been compared with the RT-PCR method with 70 clinical specimens, demonstrating that the relative accuracy is 94.3%.

#### 2.2.2. Real-Time RT-PCR

Nucleic acid extraction was automated using the ExiPrep™48 Dx instrument (Bioneer, Republic of Korea) and the ExiPrep™48 Viral DNA/RNA kit (Bioneer, Republic of Korea), which employ magnetic particle-based purification to ensure reproducibility and efficiency. Amplification was performed using the ExiCycler™96 instrument (Bioneer, Republic of Korea) and AccuPower Diarrhea V1&V2 Real-Time RT-PCR kit (Bioneer, Republic of Korea), with data analysis conducted using ExiStation™ 48 software. The entire process, including extraction (1 h) and amplification, required approximately 3.5 h.

All testing and interpretation were carried out according to the manufacturers’ instructions. Briefly, sample preparation involved transferring collected stool samples to sample tubes and loading them into cartridges. Nucleic acid extraction (loading the cartridge and accessories into the ExiPrep^TM^48 Dx) was performed by setting up and running the instrument. Once extraction was completed, samples were prepared for RT-PCR (sealed, vortexed, and then spun using ExiSpin^TM^). Finally, Real-Time PCR was performed (automatically amplifying the extracted samples) and results were analyzed using ExiStation^TM^ 48 software.

AccuPower Diarrhea V1&V2 Real-Time RT-PCR (containing two premix type kits; DV1 and DV2) is an easy-to-use, pipetting-free (since all components for the assay are contained within a tube) multiplex diagnostic kit for the simultaneous detection of six different acute diarrhea-causing viruses (rotavirus, adenovirus, norovirus genogroups I and II, astrovirus, and sapovirus) from human fecal samples. ExiPrep^TM^48 Viral DNA/RNA and Bioneer AccuPower Diarrhea V1&V2 Real-Time RT-PCR kits are vacuum-dried premix-type, which makes them easy to use, preserves the overall activity of the mixed reagents, and maximizes reproducibility.

#### 2.2.3. Statistical Analyses

Statistical analyses were conducted to compare the performance of the CIA and RT-PCR methods. Positive percent agreement (PPA), negative percent agreement (NPA), and overall percent agreement (OPA) were calculated, along with Cohen’s kappa coefficient, to assess agreement beyond chance. Interpretation of kappa values followed the Bland and Altman guidelines, with values below 0 indicating no agreement and 0.01–0.20 indicating slight agreement. Statistical analyses, including 95% confidence intervals (CIs), were performed using MedCalc version 17.6. For astrovirus and sapovirus, which were not tested using CIA methods, RT-PCR results were analyzed independently, and prevalence was reported descriptively.

## 3. Results

### 3.1. Demographics

A total of 63 stool samples from patients presenting with diarrhea were analyzed in this study. The demographic characteristics of the study population are summarized in Table 1. Female patients accounted for the majority (67%), and most samples (78%) were collected from hospitalized patients. Nearly half of the patients (49%) were children, with 32% under the age of five. All patients had diarrheal stool, whether it was clearly stated as a working diagnosis or not. Eleven percent of the patients were immunocompromised.

### 3.2. CIA and RT-PCR

The comparison of chromatographic immunoassay (CIA) and real-time reverse transcription polymerase chain reaction (RT-PCR) for detecting gastrointestinal viruses and agreement metrics are presented in Table 2.

For norovirus, RT-PCR identified nine positive samples, of which only one was concurrently identified by CIA. CIA missed eight samples, while three CIA-positive samples were negative by RT-PCR. The positive percentage agreement (PPA) for norovirus was 25%, with an overall percentage agreement (OPA) of 82.54% and a negative percentage agreement (NPA) of 86.4%. According to clinical state, one false positive CIA result was from a patient with noninfectious diarrhea and two from patients with infectious diarrhea. The only concomitant positive result (CIA and RT-PCR) was from a patient with infectious diarrhea; however, this specimen was also falsely CIA-positive for rotavirus and adenovirus. The remaining two positive RT-PCR results, missed by CIA, were from patients involved in a school outbreak of diarrhea and vomiting.

For adenovirus, no samples were identified as positive by RT-PCR, whereas CIA identified 12 false positives. This resulted in an OPA of 80.95%, with an NPA of 100%, although PPA could not be calculated due to the absence of true positives. Four false positive CIA results were from patients with noninfectious gastroenteritis, one from an immunocompromised patient, and the remaining seven results were from patients with infectious diarrhea.

Rotavirus detection showed one true positive sample identified by both RT-PCR and CIA (from the patient with noninfectious gastroenteritis), while ten samples were false positives by CIA (four from patients with noninfectious gastroenteritis and six from patients with infectious diarrhea). The PPA for rotavirus was 90.9%, with an OPA of 84.13% and an NPA of 100%.

For sapovirus and astrovirus, RT-PCR detected one sapovirus-positive sample, marking the first reported identification of this virus in human stool samples in Croatia. The testing was repeated twice on the same specimen with consistent results. No astrovirus-positive samples were identified by RT-PCR, and neither virus was tested using CIA due to the unavailability of compatible assays.

### 3.3. Discussion

Accurate and timely identification of viral pathogens is critical for guiding patient management, implementing infection control measures, and informing public health strategies. To our knowledge, this is the first study to compare the performance of the Bioneer AccuPower Diarrhea V1&V2 RT-PCR with a chromatographic immunoassay for detecting major gastrointestinal viruses in stool specimens. The RT-PCR multiplex method, when paired with advanced nucleic acid extraction systems like the ExiPrep™48 Dx, Bioneer, offers a simple, automated, and user-friendly solution for pathogen detection in clinical settings. Our results highlight significant discrepancies between the two methods, emphasizing the diagnostic limitations of CIA and the potential advantages of RT-PCR in specific clinical (e.g., immunosuppression) and diagnostic scenarios (e.g., cross-reactivity in CIA).

The overall findings demonstrate the superior sensitivity and specificity of RT-PCR compared to CIA, particularly for norovirus and adenovirus detection. For norovirus, CIA had a low PPA of 25%, indicating that it missed 75% of norovirus-positive cases identified by RT-PCR. Additionally, the observed false positive rate with CIA highlights its limited reliability for this pathogen. These results align with the existing literature, suggesting that norovirus detection is challenging with rapid antigenic tests, especially in low-viral-load cases or during outbreaks, where accurate results are critical for infection control and management.

Adenovirus detection by CIA showed poor agreement with RT-PCR, with 12 false positives reported and a PPA of 0%. This lack of sensitivity raises concerns about the clinical utility of CIA for diagnosing adenovirus gastroenteritis, particularly in immunocompromised patients, where timely and accurate identification is crucial. While the NPA for adenovirus was 100%, the false positives undermine CIA’s reliability as a standalone diagnostic tool.

Rotavirus detection presented a relatively better performance with CIA, achieving a PPA of 90.9% and an OPA of 84.13%. However, the presence of false positives (15.8%) highlights the need for confirmatory testing, especially in cases where clinical presentation does not align with CIA results. The higher agreement observed for rotavirus suggests that CIA may still hold some value in resource-limited settings for this pathogen, but its results should be interpreted cautiously.

In addition, it is important to mention that six samples gave a simultaneous positive result for rotavirus and adenovirus (all were RT-PCR-negative), and four samples gave a positive CIA result for all three viruses tested (of which only one was RT-PCR-positive for norovirus).

The detection of sapovirus in a stool sample using RT-PCR in Croatia represents the first documented evidence of this pathogen in human specimens in the region. The infection occurred in a young girl, aged 8, who presented with watery diarrhea. This discovery emphasizes the evolving global importance of sapovirus, which has emerged as a significant cause of acute gastroenteritis, particularly in young children, following the reduction in rotavirus cases due to vaccination. Globally, the pooled prevalence of sapovirus in AGE cases is estimated at 3.4%, rising to 5.6% when more sensitive RT-qPCR assays are employed, showcasing the advantages of molecular diagnostics in pathogen identification. While sapovirus often causes mild, self-limiting gastroenteritis, severe cases requiring hospitalization and complications such as dehydration have been reported, especially in low-resource settings. Unlike other regions with established data, this first report underscores the importance of continued surveillance in underrepresented countries like Croatia, particularly as molecular diagnostic tools facilitate the detection of emerging or previously underreported pathogens [29].

Similarly, no positive cases of astrovirus were identified in this study, which underscores the potential role of multiplex RT-PCR panels in expanding the spectrum of detected viruses and enhancing our understanding of their epidemiology.

The manufacturers of the norovirus CIA acknowledge the potential for false positive results in children under 1 year of age, who are often the primary target population for this test. This limitation further underscores the recommendation to opt for the multiplex PCR method instead, which offers greater accuracy [30].

The manufacturer of a rapid antigen test for adenovirus and rotavirus claims an accuracy of over 97.0% overall agreement (OA), based on a comparison with the latex agglutination method. On the contrary, the results of our study have showed a significantly lower percentage of agreement when RT-PCR is used instead of a rapid antigenic test as a comparator. However, we believe that the accuracy of rapid antigen tests should ideally be compared to viral cultures or, when cultures are unavailable, at least to molecular methods.

These findings emphasize the limitations of rapid antigen-based tests, particularly their low sensitivity and potential for false positives. Stool samples are inherently challenging for antigen detection due to variability in sample consistency, the presence of interfering substances, and the rapid degradation of viral antigens. RT-PCR, with its superior sensitivity and specificity, offers a more reliable alternative for detecting gastrointestinal viruses.

Despite the clear advantages of RT-PCR, its high cost and longer turnaround time compared to EIA/CIA may limit its routine application in all settings. A two-step diagnostic approach, starting with initial CIA screening followed by confirmatory RT-PCR, could balance cost-effectiveness with diagnostic accuracy. In UHS, three gastrointestinal viruses (rotavirus, adenovirus, and norovirus) are routinely tested with CIA, which is cost-effective for our institution. However, if all gastrointestinal viruses were tested with antigenic tests, perhaps not only the diagnostic accuracy but also the cost-effectiveness would favor RT-PCR.

This study is limited by the lack of a gold standard reference method, such as viral culture, to establish the true sensitivity and specificity of the assays. Future research should include larger sample sizes and comparisons with other molecular diagnostics to validate the performance of RT-PCR. Additionally, the interpretation of multiplex RT-PCR results in mixed infections warrants further investigation, as distinguishing between true pathogens and incidental findings remains a challenge.

## 4. Conclusions

In conclusion, RT-PCR demonstrates clear advantages over CIA for detecting gastrointestinal viruses, particularly in terms of sensitivity and reliability. The incorporation of molecular diagnostics into routine laboratory workflows, especially for specific clinical scenarios such as outbreaks or immunocompromised patients, should be strongly considered. Finally, the detection of sapovirus in Croatia underscores the importance of RT-PCR in identifying emerging pathogens and broadening our understanding of viral gastroenteritis.

## Figures and Tables

**Table 1:** Demographics of the study population (N = 63).

Patient Data	No. (%) of Patients
Gender	
Male	21 (33%)
Female	42 (67%)
Age (years)	
0–≤5	20 (32%)
>5–≤18	11 (17%)
>18	32 (51%)
Location	
Hospital ward	49 (78%)
Outpatient clinic	14 (22%)
Working diagnosis and underlying condition	
Infectious diarrhea in immunocompetent patients	30 (48%)
School outbreak of diarrhea and vomiting	14 (22%)
Noninfectious gastroenteritis and colitis	12 (19%)
Diarrhea in immunocompromised patients	7 (11%)

**Table 2:** Human stool co-testing with CIA and RT-PCR.

Target	CIA*/PCR^†^ −/−	CIA*/PCR^†^ +/−	CIA*/PCR^†^ −/+	CIA*/PCR^†^ +/+	PPA^‡^ (95% CI)	NPA^§^ (95% CI)	OPA^||^ (95% CI)	Cohens’ Kappa
Adenovirus	51	12	0	0	0	100.0	80.95 (0.000)	0.000
Rotavirus	52	10	0	1	90.9	100.0	84.13 (−0.109 to 0.392)	0.142
Norovirus	51	3	8	1	25.0	86.4	82.54 (−0.203 to 0.348)	0.072
Sapovirus	NA/62	NA	NA	NA/1	/	/	/	/
Astrovirus	NA/63	0	0	0	/	/	/	/

Note: CIA* (chromatographic immunoassay): JustChek Norovirus; AcroBiotech Rota/Adenovirus; PCR^†^: Bioneer AccuPower Diarrhea V1&V2 Real-Time RT-PCR; PPA^‡^ (positive percent agreement), NPA^§^ (negative percent agreement), OPA^||^ (overall percent agreement).

## Data Availability

The data presented in this study are available on request from the corresponding author due to the privacy.
